# Joint analysis of interaction and psychological characteristics in english teaching based on multimodal integration

**DOI:** 10.1186/s40359-024-01585-0

**Published:** 2024-03-04

**Authors:** Chao Li

**Affiliations:** School of Culture and Education, Shaanxi University of Science & Technology, 710021 Xi’an, Shaanxi China

**Keywords:** Multimodal fusion, English teaching, Interactive teaching, Psychological feature, Interactive research

## Abstract

The intersection of psychology and English teaching is profound, as the application of psychological principles not only guides specific English instruction but also elevates the overall quality of teaching. This paper takes a multimodal approach, incorporating image, acoustics, and text information, to construct a joint analysis model for English teaching interaction and psychological characteristics. The novel addition of an attention mechanism in the multimodal fusion process enables the development of an English teaching psychological characteristics recognition model. The initial step involves balancing the proportions of each emotion, followed by achieving multimodal alignment. In the cross-modal stage, the interaction of image, acoustics, and text is facilitated through a cross-modal attention mechanism. The utilization of a multi-attention mechanism not only enhances the network’s representation capabilities but also streamlines the complexity of the model. Empirical results demonstrate the model’s proficiency in accurately identifying five psychological characteristics. The proposed method achieves a classification accuracy of 90.40% for psychological features, with a commendable accuracy of 78.47% in multimodal classification. Furthermore, the incorporation of the attention mechanism in feature fusion contributes to an improved fusion effect.

## Introduction

There is a close relationship between psychology and English teaching, any excellent English teacher cannot do without the understanding and in-depth study of psychology. As an important branch of psychology, the learning psychology is one of the theoretical foundations of English teaching method, which mainly studies the psychological process and mechanism of how students acquire English knowledge, skills and develop intelligence (Picard, 2016) [1]. In practical teaching, English teachers attach importance to the study of students’ learning psychological activities. The psychology is often used to guide specific teaching English teaching, which can continuously improve the quality of English teaching (Warsi & Khurshi, 2022) [2]. The student period is a very important period for the formation of personality and the accumulation of knowledge, and their physical and psychological states are in the growth stage. At this stage, children are extroverted and imitative, so the teachers should make use of these characteristics to make them always maintain strong interest in learning (He, 2020) [3]. With the increase of age and learning content, students’ psychological activities also change, their interest in learning mechanical imitation and intuitive explanation gradually weakened. Therefore, it is an important problem for English teachers to master students’ psychological characteristics and combine them with English teaching to organize English teaching activities (Wang, 2021) [4].

Students of different ages have different physiological and psychological characteristics, so English teaching should be organized with emphasis in English teaching. It is very important to guide students to consciously carry out interactive practice, which can cultivate students’ ability to use language independently (Huang, 2020; Ding, 2020) [5–6]. Therefore, the analysis of students’ psychological characteristics based on the interaction of English teaching is of great significance, which can improve the quality of English teaching. Human emotions are composed of multimodal information, and the information between each mode is complementary, so there are some inevitable shortcomings in psychological counseling through a single mode. In order to improve the accuracy of psychological feature recognition, scholars propose to fuse the information of multiple modes, which can identify psychological features more comprehensively and accurately (Zhang et al., 2022; Huang, 2010) [7–8]. The multimodal emotion recognition is a new research direction in the NLP, which mainly uses deep neural network to process emotion features (Geetha, 2024) [9]. For multimodal emotion recognition task, scholars often use deep network model to realize the fusion of multimodal features, and the emergence of neural network model has also led to the development of multimodal emotion recognition. By establishing various neural network models and using multimodal data for end-to-end training, excellent results have been achieved in the field of emotion recognition.

Traditional English teaching primarily centers on imparting language knowledge, but the advent of modern technology has ushered in new possibilities for instructional interaction, such as online learning platforms and virtual reality technology. Furthermore, students exhibit diverse psychological characteristics during the learning process, encompassing aspects like learning motivation, attention, and emotion, which significantly influence learning outcomes. Thus, this study endeavors to enhance the effectiveness of English learning through multimodal fusion. By jointly analyzing students’ psychological characteristics, the aim is to gain a profound understanding of their needs and challenges in learning, paving the way for personalized teaching support.

This research not only broadens the application scope of educational technology in English teaching but also serves as a reference for other disciplines, fostering the advancement of educational technology. In terms of motivation, the study prioritizes addressing students’ diverse learning needs, leveraging technology development to create a more engaging and interactive learning environment, ultimately enhancing students’ participation and interest in learning. Through experimental design, questionnaire surveys, and educational technology development, the researchers seek to validate the efficacy of multimodal integration in English teaching. Additionally, the study aims to unravel the intricate relationship between students’ psychological characteristics and learning performance, offering crucial theoretical and practical insights for the future development of educational technology and the optimization of English teaching methodologies.

In the domain of English teaching, the nexus between instructional interaction and psychological traits is garnering increasing attention. The principal challenge in this paper revolves around the imperative to formulate a resilient framework for the optimal integration of diverse modalities, including linguistic, visual, and emotional cues. The proposed solution seeks to enhance the efficacy of feature integration by refining the existing multimodal fusion mechanism, with a specific focus on improving the precision of emotion recognition. The adoption of a multimodal fusion approach presents an avenue to bridge existing gaps in comprehending the nuanced interplay between pedagogical interactions and psychological traits within the context of English language education.

This paper introduces a novel model for the joint analysis of interactive features and psychological characteristics in English teaching, focusing on multimodal fusion. We comprehensively consider three modalities: image, acoustics, and text, with the aim of enhancing teaching effectiveness.

Firstly, we use multimodal signals to detect emotions in videos, utilizing image, acoustic, and text modal data as inputs to the model. By employing different neural networks to extract feature vectors for each modality, we can capture information more comprehensively.

Next, to facilitate effective cross-modal feature fusion, we introduce a cross-modal attention mechanism proposed in this paper. This mechanism enables interaction between source and target modalities, effectively promoting information exchange and integration. By merging feature vectors from different modalities, we obtain richer and more representative features.

Finally, we process the fused features through a fully connected network to obtain the ultimate prediction results. This seamless integration of multimodal information provides a fresh perspective on the analysis of interactivity and psychological characteristics in English teaching. The proposed approach and model are expected to make significant strides in the field of education, contributing to improved teaching effectiveness and personalized education.

## Related work

### The interactive research on english teaching

The interaction is the core of classroom teaching, which has an important impact on teaching effect and teaching quality. The effective classroom interaction can stimulate the learning interest of students, which can improve the learning efficiency of students and promote the occurrence of deep learning. The interaction in English teaching refers to the interaction between people, the interactive objects mainly include teachers, auxiliary teachers, lecturers and remote students (Chen, 2017; Wu, 2020) [10–11]. This paper mainly focuses on teacher-student interaction, which mainly includes two parts, one is the interaction between the lecturer and the students at both ends, and the other is the interaction between the auxiliary teacher and the remote students. The teacher factors influencing the interaction mainly include teaching concept, knowledge structure, teaching preparation, equipment use, and feedback mode, and the student factors influencing the interaction mainly include learning attitude, peer pressure, cooperative learning and environmental adaptation (Cao, 2014) [12]. The main problem of teacher-student interaction is the single form of interaction, the frequency of interaction between lecturers and lecturers is significantly higher than that between lecturers and remote students in traditional English teaching (Lin, 2018) [13]. The interaction of English teaching is affected by many factors, and its influence on teaching effect is difficult to reflect. The organic combination of English teaching interaction and the psychological characteristics of students can help to analyze the psychological characteristics, which is beneficial to improve the quality of English teaching.

The research on the interactivity of English teaching involves various aspects at the psychological level, focusing on students’ cognitive, emotional, and motivational dimensions. On the cognitive level, studies indicate that excessive cognitive load in teaching may impact students’ interactivity, necessitating the design of appropriately challenging tasks based on students’ cognitive levels (Chew and Cerbin, 2021) [14]. Simultaneously, considering students’ diverse learning styles, a variety of teaching methods can meet their cognitive needs, enhancing learning outcomes. On the emotional level, psychological research underscores the importance of providing emotional support in teaching. Positive learning emotions contribute to fostering students’ positive attitudes towards English learning, thereby enhancing their interactivity (Tremblay et al., 2021) [15]. Additionally, creating a positive and supportive learning atmosphere is crucial, making students more willing to engage in interactions, share opinions, and experiences (KEE, 2021) [16]. On the motivational level, students’ autonomous learning motivation plays a critical role in interactivity. By stimulating students’ interest and autonomy, teaching can enhance their interactivity in English learning (Challob, 2021) [17].

Clearly defined learning objectives are also an effective means to increase students’ motivation and participation (Bai et al., 2020) [18]. On the social level, peer interaction significantly influences psychological characteristics. Teaching design should encourage collaboration and communication among students to promote social interaction. Furthermore, fostering positive teacher-student relationships contributes to building students’ trust and a sense of security in teaching, leading to more active participation in learning interactions (Munir et al., 2021) [19].

In conclusion, by addressing cognitive, emotional, motivational, and social psychological dimensions, educators can propose more targeted teaching strategies and methods, creating a positive learning environment and inspiring students’ initiative and enthusiasm for learning English. This contributes to promoting effective interactivity in English teaching, enhancing students’ learning experiences and outcomes.

### The psychological feature analysis based on multimodal fusion

The multimodal fusion can make full use of complementary information in multi-modal data, which has obvious advantages for multi-class classification tasks, and different multimodal fusion methods can be applied to different scenarios. At present, the multi-modal fusion method is applied in different scenarios such as visual question answering, behavior recognition, image subtitle generation, emotion recognition, and emotion analysis, and the fusion methods are also very different in different application scenarios (Zhang, 2019; Lu, 2023) [20–21]. The multimodal emotion recognition has always been an important branch of emotion computing, and the multimodal emotion recognition has attracted more and more researchers to explore in recent years (Lu, 2016) [22]. A context-aware multi-modal emotion recognition algorithm is also proposed, which mainly combines three aspects of context relations, one is emotion recognition based on multiple modes, the second is the use of CNN based on self-attention mechanism to encode these information, and the third is the construction of the model (Fisher, 2012) [23]. In the current psychological feature recognition, the introduction of attention mechanism cannot significantly improve the model training performance. The LSTM and tensor-based convolution network are used to dynamically model the intra-modal and inter-modal interactions, and good results are achieved in emotion classification.

Some approaches employ techniques such as feature concatenation, addition, multiplication, or attention mechanisms to integrate multimodal information. Huang et al. (2019) [24] proposed a method for deep multimodal attention fusion, called Deep Multimodal Attention Fusio, for emotion analysis in images and text. This method outperformed state-of-the-art baseline models on four real datasets. Truong et al. (2019) [25] introduced a visual attention network, suggesting that in this task, visual information assists textual information. Specifically, it does not independently express emotions but can emphasize certain entities in the text. The model uses visual information as an alignment method, utilizing attention to highlight important sentences in the document for emotion classification. Han et al. (2021) [26] extracted complementary information between modalities based on attention mechanisms and improved unimodal representations through gate control mechanisms. Additionally, Zhou et al. (2021) [27] proposed a multimodal polarity prediction network, performing matrix multiplication on representations of images, text, and aspect features to achieve multimodal interaction. Zhang et al. (2021) [28] employed a pair of memory networks to capture intra-modal information and extract interaction information between different modalities, then designed a discriminative matrix to supervise the fusion of modal information. Gu et al. (2021) [29] designed an Attention Capsule Extraction and Multi-Head Fusion Network for aspect-level multimodal sentiment classification. Through the integration of multi-head attention mechanisms and capsule networks, it captures interactions between multimodal inputs. Existing models tend to leverage the overall information from images, overlooking some local features, such as facial emotions, as visual emotional cues (Yang et al., 2022) [30].

## The joint analysis of english teaching interaction and psychological characteristics based on multimodal fusion

It is necessary to determine the overall framework of the model before constructing a joint analysis model of English teaching interactivity and psychological characteristics based on multimodal fusion. In this paper, an improved multi-modal fusion mechanism is proposed to enhance the effectiveness of fusion features, which has higher recognition accuracy for emotion recognition. The model proposed in this paper realizes the feature fusion of the three modes of image, acoustics and text, the attention mechanism is used for feature fusion, and the fusion feature is predicted by psychological characteristics finally.

### The overall process of the model

**Fig. 1:**
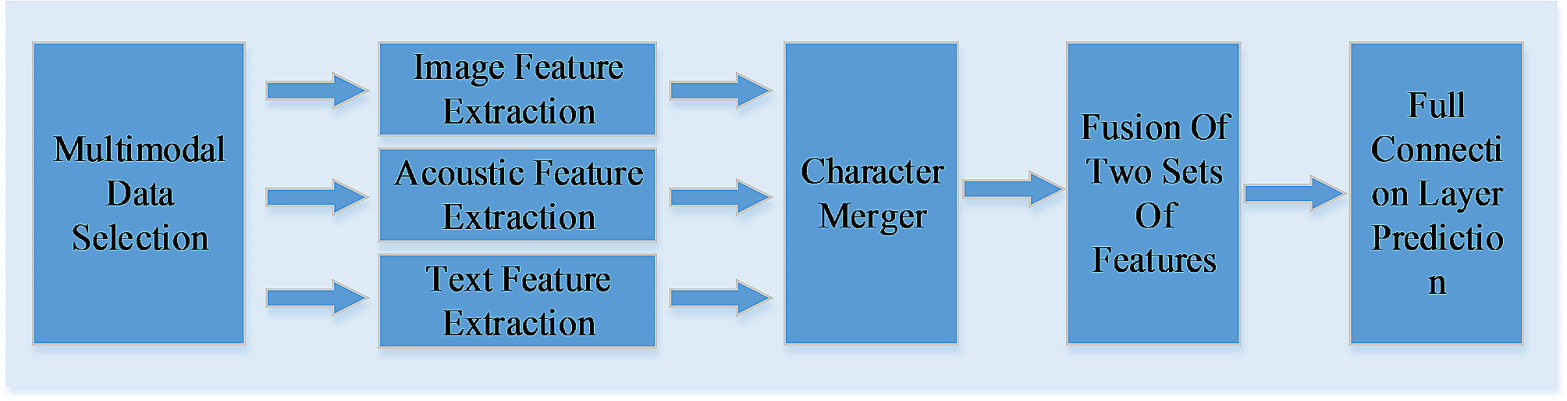
The overall process of the model

In the multimodal emotion recognition task, the emotional state of a video may be different in different periods, and the emotion of different modes may be different at the same time. Therefore, in order to achieve more effective feature fusion, the learning interactive information between modes is a key problem. In current research, the fusion strategy based on attention mechanism has achieved good results. The purpose of this paper is to realize the research of emotion recognition by using the method of cross-modal attention mechanism, which can make the multi-modal data of images, acoustics and text feature fusion. The overall process of the model is divided into four steps, as shown in Fig. 1.

Firstly, the multi-modal signals are used to detect emotions in video, and three kinds of modal data are used as input of the model, including three low-level feature sequences from images, acoustics and text. In view of the different characteristics of each modal feature, different neural networks are used to extract features. After obtaining different modal feature vectors, the text information is transmitted to the acoustic information, and the acoustic information is transmitted to the visual information, so that the source mode and the target mode interact to realize the cross-modal feature fusion (Song, 2019) [31]. The attention between the two modal features is learned through the network, and the low level feature of the source mode is used to repeatedly strengthen the target mode. Then, the output results are spliced to obtain the fusion characteristics, and the prediction results are finally obtained through the full connection network.

### The data preprocessing

Multimodal information, characterized by the integration of various data modalities such as text, images, and sound, is known for its ability to provide a richer and more comprehensive understanding of content. However, the challenge lies in effectively fusing these modalities to enhance the overall information processing. In order to address this challenge, a methodical approach is proposed, focusing on the temporal aspect of the multimodal content.

The first step in this proposed method involves the extraction of timestamps for each word in the given content, encompassing both the start and end times. This temporal segmentation allows for a more granular analysis of the content, enabling the alignment of textual elements with corresponding visual and acoustic cues. These timestamps serve as the foundation for subsequent multimodal processing.

Following the acquisition of timestamps, the discourse is systematically divided into video clips based on the temporal boundaries identified earlier. This segmentation aligns with the temporal distribution of words, creating smaller units for more targeted feature extraction. Each video clip encapsulates a specific timeframe of the discourse, providing a localized context for the subsequent analysis.

The next critical step in the proposed method involves feature extraction from the video clips. By leveraging the temporal alignment established through timestamps, the visual and acoustic features at the word level are extracted and averaged. This process, inspired by the work of Kalra (2020) [32], ensures that the multimodal features are captured in a synchronized manner, enhancing the cohesiveness of the extracted information.

The averaging of features at the word level serves to mitigate redundant information between modalities while preserving the complementary nature of multimodal data. This nuanced approach allows for a more efficient fusion of visual and acoustic features, overcoming the challenges posed by potential redundancies.

### The joint analysis model based on multimodal fusion

#### The single mode feature extraction

In order to solve the problem of insufficient information in single modal psychological feature recognition, this paper uses multi-modal fusion method to synthesize image, acoustic and text feature information. Firstly, this paper defines the multimodal data, as shown in Formula 1.

1 $$X=\left\{{Ti, Si, Vi}\right\}$$

Where ${T_i}$ represents the text sequence, ${S_i}$ represents the acoustic sequence, and${V_i}$ represents the image sequence.

The text data are different from the processing of image, and the text-related issues often use the full text information. The LSTM network has two advantages in processing sequential data, one is that it can globalize the data, and the other is that it has memory units. The global information with context dependence can be obtained by processing data with temporal or spatial correlation properties, then the memory unit controls the retention of key historical information, which can achieve long-term and short-term memory function. Therefore, this paper uses the LSTM network to capture the feature information of context clues, and the model uses full-word mask and word mask to train. The word mask is the most intuitive mask method, and the full-word mask is based on words. In this paper, one-way long short-term memory network is used to capture the time feature, and the text feature vector is calculated as shown in Formula 2.

2 $${t_i}=sLSTM\left\{ {{T_i},{\theta _i}^{{lstm}}} \right\}$$

Where ${T_i}$ represents the text sequence, ${t_i}$ represents the text feature quantity.

For acoustic features, the corresponding tools are used to process audio, which can convert each acoustic file into digital. Due to the great randomness of acoustics and the correlation between adjacent sequences, some scholars use the time-domain convolution network to model the long-term data, and the time-domain convolution network has strong advantages in model size and convergence rate compared with other neural network models (Zhang, 2018) [33]. Due to the limitation of the receptive field, the time-domain convolution network cannot complete the global modeling. Therefore, this paper also uses the LSTM network to extract the acoustic mode feature, and the obtained acoustic feature vector is calculated as shown in Formula 3.

3 $${s_i}=sLSTM\left\{ {{S_i},{\theta _i}^{{lstm}}} \right\}$$

Where ${S_i}$ represents the acoustic sequence, and ${s_i}$ represents the acoustic characteristic quantity.

For image features, it is necessary to segment the image to obtain each image with key feature information, and then take the face image in all image sequences as the output information of acoustic mode. Since CNN has a strong performance in the field of image recognition, many image recognition models are constructed based on CNN. Considering the size of the input image data and the performance of the experimental equipment, this paper uses multi-scale convolutional neural network to extract features from image input more effectively. The calculation is shown in Formula 4–5.

4 $${G_\alpha }(V,\theta )=\left\{ {{V_\alpha }\left| {\alpha \in \Omega } \right.} \right\}$$

5 $$H(x)=F(x)+I(x)$$

Where V represents the input feature, $\Omega$ represents the kernel set under the visual mode, and $H(x)$ represents the final output of the residual module.

#### The multimodal emotion analysis based on attention mechanism

In order to integrate multiple feature information, a multi-feature fusion module is designed in the network, which is composed of similar information calculation and multi-head attention mechanism. This module focuses on adding similar information between different modalities, effectively fusing text and visual related features, and obtaining a more expressive and comprehensive multi-modal feature representation.

**Fig. 2:**
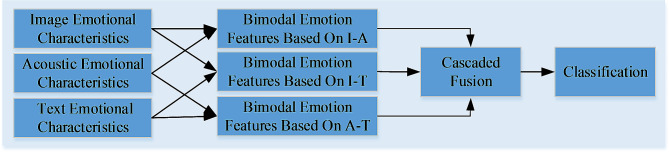
The multimodal emotion analysis based on attention mechanism

The attention mechanism based sentiment analysis model is proposed in this paper, in which the attention mechanism is introduced to effectively fuse the emotional features. For the extracted emotion features, we need to obtain the single-modal emotion features through the self-attention mechanism, and then obtain the mutual attention emotion features between different modes through the combination of two modes. Finally, the emotional features are cascaded and fused to obtain the complete multimodal emotional features, which are then sent to the classifier for emotional discrimination. The specific multimodal emotional analysis based on attention mechanism is shown in Fig. 2.

After obtaining the image self-attention emotion feature, acoustic self-attention emotion feature and text self-attention emotion feature, the mutual attention emotion feature of different modal interaction can be obtained. The obtained mutual attention emotion features can be cascaded and fused to obtain complete multimodal emotion features, and then the multimodal emotion features are sent to the classifier for emotion classification.

The input of attention mechanism is the acquired emotional characteristics, and the attention mechanism adds importance weights to different modes mainly through the interaction between two different modes. Taking the combined image-acoustics as an example, the attention mechanism can obtain the features that need to be focused in relative image and acoustic emotional features. These features that need to be focused need to be assigned higher probability weights, and those with lower attention need to be assigned lower probability weights.

The input of image-acoustic mutual attention mechanism is image self-attention emotional characteristics and acoustic self-attention emotional characteristics, and the image-acoustic mutual attention emotional characteristics obtained by mutual attention mechanism are shown in Formulas 6–8.

6 $${F^{V - S}}=soft\hbox{max} \left( {\frac{{{F^{{V_1}}}{{\left( {{F^{{S_1}}}} \right)}^T}}}{{\sqrt d }}} \right){F^{{V_1}}}$$

7 $${F^{S - V}}=soft\hbox{max} \left( {\frac{{{F^{{S_1}}}{{\left( {{F^{{V_1}}}} \right)}^T}}}{{\sqrt d }}} \right){F^{{S_1}}}$$

8 $${F^{VS}}=concat\left( {{F^{V - S}},{F^{S - V}}} \right)$$

Where ${F^{V - S}}$ represents the important weight feature added by image emotion feature relative to acoustic emotion feature, ${F^{S - V}}$ represents the important weight feature added by acoustic emotion feature relative to image emotion feature, ${F^{VS}}$ Represents image-acoustic emotional features.

Finally, the complete multimodal sentiment features can be obtained by cascade fusion, as shown in Formula 9.

9 $$F=concat\left( {{F^{VS}},{F^{VT}},{F^{ST}}} \right)$$

Where ${F^{VS}}$ represents the image-acoustic emotion feature, ${F^{VT}}$ represents the image-text emotion feature, and ${F^{ST}}$ represents the acoustic-text emotion feature.

Finally, the bi-modal emotion features are spliced and fused by cascade fusion, the multimodal emotion features can be obtained, and the classifier is used for emotion classification.

## Experiments and analysis

### Data set

To assess the efficacy of the proposed psychological feature recognition model, training and testing were conducted using public datasets. It’s worth noting that progress in emotional analysis within the domestic landscape has been relatively slow. Mainstream sentiment analysis libraries in China primarily focus on single-modal or dual-modal sentiment analysis, lacking a three-modal sentiment database that integrates expression, acoustics, and text disclosure.

The datasets employed in this study are sourced from the Multimodal Language Analysis in the Wild: CMU-MOSEI Dataset and Interpretable Dynamic Fusion Graph, representing the largest three-modal dataset available. The CMU-MOSEI dataset is characterized by multi-label features, implying that each sample may correspond to more than one emotion, and the emotions associated with each sample can vary. This dataset provides a comprehensive foundation for evaluating the proposed psychological feature recognition model.

### Evaluation index

In order to verify the effect of the experimental model, it is necessary to develop a reasonable evaluation index. For this experiment, the accuracy and F1 score are used to measure the performance of the model, and the calculation of the accuracy is shown in Formula 10. The greater the accuracy, the better the actual effect of the model.

10 $$Accuracy = \frac{{TP}}{{TP + FP}}$$

When the actual results are positive, TP represents that the prediction results are correct. When the actual result is negative, FP represents that the prediction result is correct.

The value of F1 is calculated based on each category, its evaluation of model performance is more comprehensive, and the calculation of F1 score is shown in Formulas 11–13. Among them, the precision refers to the proportion of the predicted correct samples, and the recall refers to the ratio of the correctly retrieved samples to the actual samples.

11 $$\Pr ecision{\text{=}}\frac{{TP}}{{TP+FP}}$$

12 $$\operatorname{Re} call=\frac{{TP}}{{TP+FN}}$$

13 $${F_1}{\text{=}}\frac{{2 \times \Pr ecision \times \operatorname{Re} call}}{{\Pr ecision+\operatorname{Re} call}}$$

When the actual results are positive, TP represents that the prediction results are correct. When the actual result is positive, FN represents the prediction result is wrong.

### Parameter settings

We implement the proposed AF-Net model based on PyTorch framework, and train and test it on NVIDIA A30 GPU computing resources. The experimental parameter Settings are shown in Table 1.

**Table 1:** The experimental parameter Settings

Parameter	Value
Dropout	0.1
batch_size	32
learning_rate	3E-5
attention_head	12
image_dim	2048
Optimizer	Adam

## The results and discussion

In this paper, the relevant parameters can be adjusted according to the actual training process, and the validity of the model can be verified by the test set. Among them, V represents image sentiment analysis, S represents acoustic sentiment analysis, T represents text sentiment analysis, V + S represents bimodal sentiment analysis based on image and acoustic, V + T represents bimodal sentiment analysis based on image and text, S + T represents bimodal sentiment analysis based on acoustic and text, and S + V + E represents multimodal sentiment analysis.

### The loss values on different datasets

**Fig. 3:**
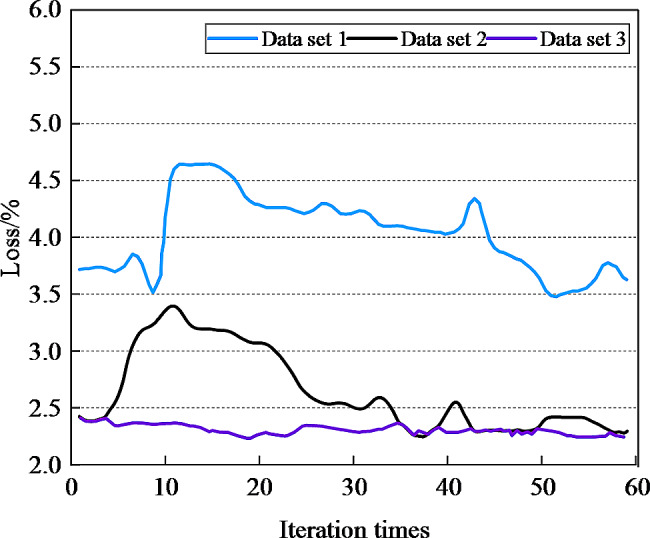
The loss values on different datasets

For regression tasks, the commonly used loss function is MAE, whose calculation takes the absolute error as the distance. The MAE is sparse, so MAE is usually added as a regular term to other loss functions as constraints. In this paper, the image information, acoustic information and text information are used to conduct the multimodal fusion English teaching psychological feature recognition experiment. The loss values on different data sets are shown in Fig. 3.

It can be seen from Fig. 3 that the loss value of dataset 1 reaches the lowest value of 3.48% when the step size is 51. When the step size of dataset 2 is 37, the loss value is the lowest, which is 2.25%. When the step size of dataset 3 is 48, the loss value is the lowest, which is 2.26%. The loss function generally refers to the error of a single training sample, in the case of no over-fitting of the model, the smaller the loss is, the closer the predicted value is to the real value. When the step size is less than 50, the loss values of the model on different data sets are less than 4%, indicating that the model in this paper not only has good performance. After fewer iterations, the model can make accurate predictions, but also can make accurate prediction after fewer iterations.

### The confusion matrix

**Fig. 4:**
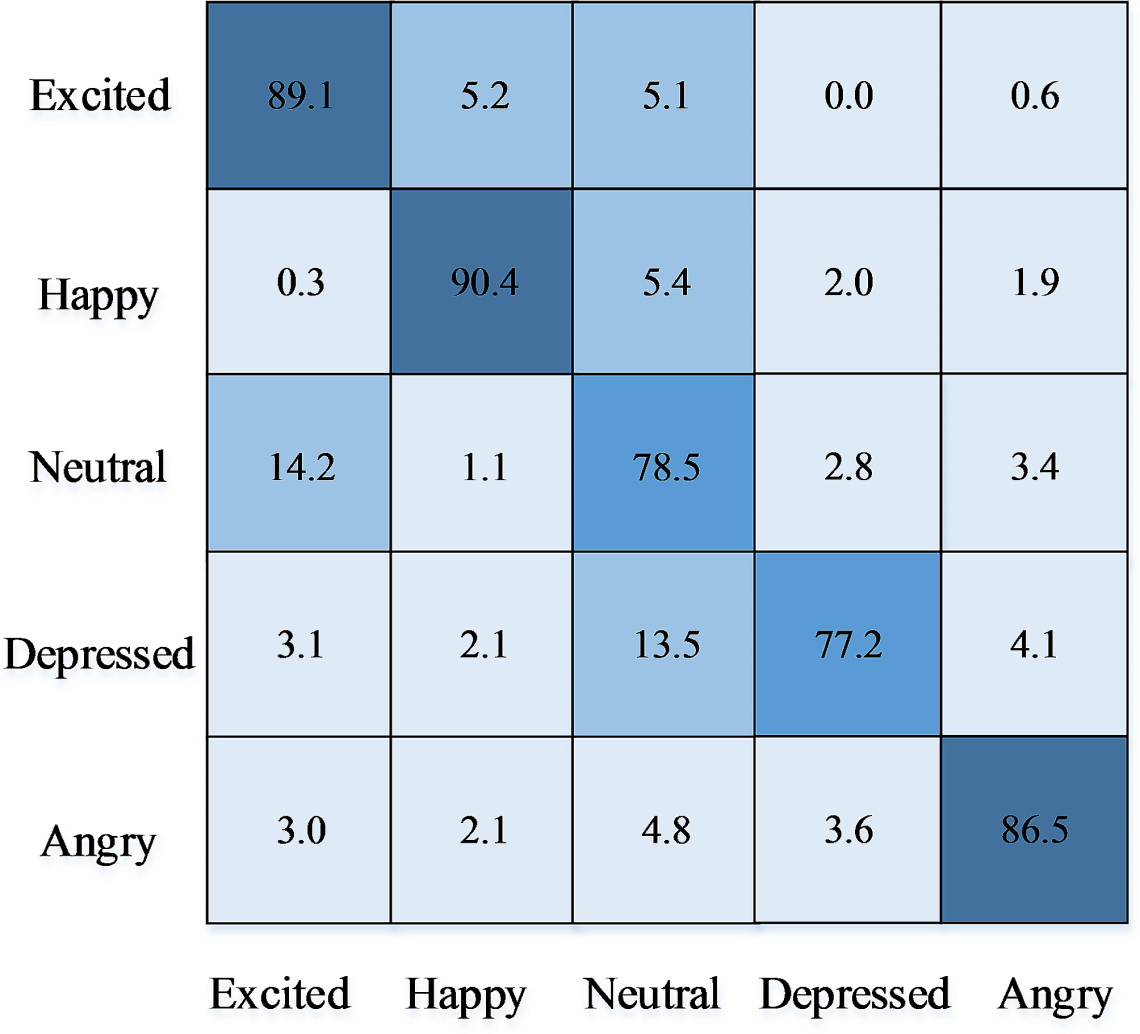
The confusion matrix

The experiment in this section mainly verifies the performance of the multi-modal psychological characteristic analysis model, and the cross-validation experiment is carried out for multi-modality. The average result after cross-validation is taken as the final result, and the confusion matrix on the data set is shown in Fig. 4. Finally, the recognition accuracy of the five psychological characteristic indexes is obtained, and the five psychological characteristic indexes represent excitement, happiness, neutrality, depression and disappointment respectively.

Examining Fig. 4 reveals the model’s adeptness in identifying five psychological characteristics, with a notable accuracy of 90.4% for happy samples. The recognition accuracy for excitement and anger samples is commendable at 89.1% and 86.5%, respectively. However, the model faces challenges in accurately identifying depression samples, resulting in the lowest accuracy, followed closely by neutral samples.

Further analysis indicates that 14.2% of neutral feature samples are misclassified as excitement samples. This misclassification is primarily attributed to the similarities in performance between neutral and happy features, causing some neutral instances to be erroneously identified as expressing excitement. Additionally, 13.5% of depression feature samples are misclassified as neutral samples. This misattribution is mainly due to the nuanced similarities between neutral and depression features, leading to instances where depression characteristics are incorrectly identified as neutral.

In summary, while the model demonstrates impressive accuracy in recognizing certain psychological characteristics, it faces challenges, particularly in distinguishing between neutral and excited states, as well as accurately identifying depression samples.

### The experimental verification results

**Fig. 5:**
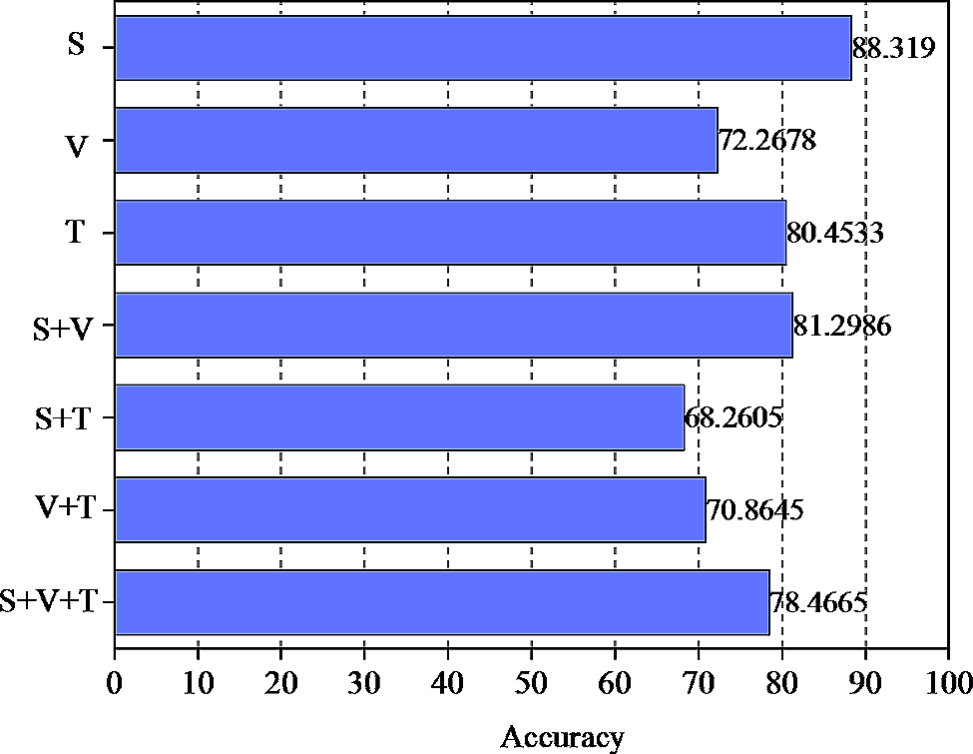
The accuracy of different models

The experiment in this section mainly verifies the performance of the multi-modal psychological characteristic analysis model, in which the cross-validation experiments are carried out for single mode, double mode and multi-mode. The average results after cross-validation are used as the final results, and the accuracy of different models is shown in Fig. 5.

As depicted in Fig. 5, in the examination of single-mode psychological characteristics, the analysis accuracy of acoustic characteristics is the highest, reaching 88.319%, followed by the analysis of text characteristics, with the analysis accuracy of image characteristics being the lowest at only 72.2678%. In dual-modal feature analysis, the dual-modal feature analysis based on image and acoustic features outperforms, and the effectiveness of each group is improved compared to the corresponding single-modal psychological feature analysis. The accuracy of psychological feature analysis based on image, acoustic, and text is 78.4665%, demonstrating a certain improvement compared to other fusion methods. This indicates the feasibility and effectiveness of the multi-modal psychological feature analysis method.

**Fig. 6:**
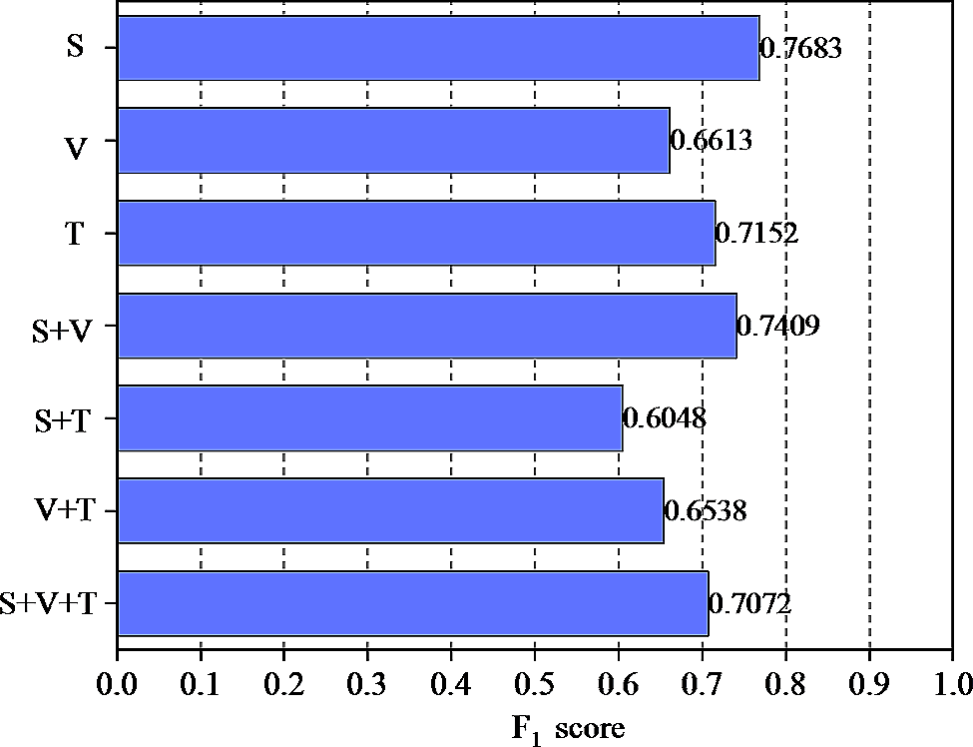
The F1 scores of different models

In the performance test of multimodal psychological characteristic analysis model, the F1 scores of different models are also tested, and the results are shown in Fig. 6.

As depicted in Fig. 6, in the examination of single-mode psychological characteristics, the F1 value for acoustic characteristics analysis is the highest, reaching 0.7683, followed by text characteristics analysis, with the F1 value for image characteristics analysis being the lowest at only 0.6613. In the investigation of dual-modal psychological characteristics, the dual-modal analysis based on image and acoustic features exhibits the most favorable outcome, with an improved F1 value for each group compared to the corresponding single-modal psychological characteristics analysis. Therefore, incorporating additional modal information alongside one modality can effectively enhance the effectiveness of psychological feature analysis. The analysis of psychological characteristics based on all three modes reveals that the F1 value can reach 0.7072, affirming the efficacy of fused three-modal psychological characteristic analysis.

### Discussion

The outcomes of this study bear significant academic implications for the field of English language teaching, particularly in the integration of emotion recognition technologies. The model’s proficiency in analyzing text characteristics, coupled with identified challenges in recognizing certain psychological states, has profound implications for enhancing language learning experiences within the English language teaching context. The demonstrated effectiveness of the model in recognizing positive emotions, such as happiness and excitement, opens avenues for the development of adaptive learning environments. Incorporating emotion recognition into educational platforms allows for tailored language learning materials and activities based on students’ emotional states, thereby optimizing engagement and motivation. This aligns with the growing interest in adaptive learning technologies that respond dynamically to learners’ affective states.

The observed outcomes in this study are underpinned by various factors influencing the model’s performance in psychological characteristic analysis. The optimization process, particularly the effect of step size, reveals a consistent alignment with established principles in machine learning, emphasizing the pivotal role of hyperparameter tuning in achieving optimal model convergence. The model’s proficiency in recognizing certain psychological characteristics, such as happiness and excitement, resonates with previous findings that highlight the distinctiveness of acoustic and text features associated with these states. However, challenges in distinguishing depression and neutral samples underscore the persistent difficulty in accurately discerning nuanced emotional states based solely on specific features, aligning with existing literature.

Modal analysis reveals intriguing insights, with the superior performance of acoustic characteristics affirming the significance of audio cues in emotion recognition. Conversely, the lower accuracy of image characteristics suggests potential complexities in visual feature extraction or dataset limitations. The success of dual-modal analysis supports the notion that combining complementary modalities enhances overall performance, consistent with established principles in multimodal studies. The effectiveness of three-modal fusion further underscores the feasibility and benefits of integrating diverse modalities, aligning with the broader literature on the advantages of multimodal approaches for comprehensive feature representation.

While the results obtained in this study may be specific to the dataset and features employed, their alignment with established theories in machine learning, emotion recognition, and multimodal analysis lends credibility to the findings. The challenges faced by the model in certain scenarios reflect persistent difficulties in the field, highlighting the need for continued refinement. Overall, the study contributes to the academic discourse by reaffirming the importance of hyperparameter tuning, showcasing the nuances in recognizing specific psychological characteristics, and supporting the efficacy of multimodal approaches in enhancing the understanding of complex emotional states.

Despite its contributions, this study is not without limitations. Firstly, the generalizability of the findings may be constrained by the specific dataset used. Variability in data sources, demographics, and cultural backgrounds could impact the model’s performance in real-world applications. Additionally, the focus on specific psychological characteristics may limit the model’s broader applicability to a comprehensive range of emotions. The study also assumes the quality and representativeness of the labeled dataset, introducing potential biases or inaccuracies in the training process. Moreover, the analysis relies on predefined features, and the effectiveness of the model may be contingent on the chosen set of features. The limitations underscore the need for diverse datasets, comprehensive feature exploration, and rigorous model evaluation in future studies.

Future research endeavors can build upon this study by addressing specific areas for improvement. Firstly, an exploration of more diverse and expansive datasets encompassing various demographics and cultural contexts can enhance the generalizability of emotion recognition models. Researchers should also consider the incorporation of additional modalities, such as physiological signals or facial expressions, to further enrich the feature space and improve model robustness.

To overcome limitations associated with predefined features, future studies could explore the application of deep learning techniques for automatic feature learning. Investigating advanced architectures, such as attention mechanisms or transformer models, may contribute to capturing more intricate patterns in emotional expressions. Moreover, a focus on interpretability and explainability of the model’s decisions is essential for real-world applications. Future researchers should explore methods to enhance model transparency, facilitating the integration of emotion recognition technologies into contexts where interpretability is paramount, such as clinical settings.

## Conclusion

In this study, we successfully constructed a multimodal fusion-based joint analysis model for English teaching interactivity and psychological characteristics. The model is designed to apply psychological principles to guide the actual English teaching process. Given the limitations of traditional single-modal recognition methods and direct cascading fusion approaches, we introduced attention mechanisms to improve multimodal fusion methods, thereby more effectively constructing an English teaching psychological feature recognition model. This model can accurately identify five psychological characteristics of learners, and the attention mechanism introduced during the feature fusion stage significantly enhances the fusion effectiveness. In comparative experiments, multimodal psychological health recognition based on images, acoustics, and text showed a clear advantage in overall accuracy and F1 scores over the results of bimodal psychological health recognition, providing strong support for the effectiveness of fusing images, acoustics, and text in sentiment analysis.

This approach not only enhances the personalization of instructional content but also furnishes educators with profound insights to respond more adeptly to individual student variations. In the practical teaching scenario, this in-depth interactive analysis contributes to refining the teaching process and enhancing students’ learning experiences and effectiveness. Consequently, by employing the multimodal fusion method in this study, we not only elevate the accuracy of interactive teaching analysis but also equip educators with more robust tools to advance personalized and emotionally engaging educational practices. This holds substantial practical significance for enhancing teaching quality and fostering students’ learning motivation.

However, despite achieving satisfactory results, we acknowledge some limitations in the research. Firstly, there is room for improvement in the model’s adaptability in specific contexts, requiring validation with more real-world scenario data. Secondly, training data may exhibit certain biases, necessitating a broader and more balanced sample to enhance the model’s generalization performance. Future research could further explore new modal fusion methods and consider introducing more dimensions of psychological features to enhance the model’s comprehensiveness and accuracy.

## Data Availability

No datasets were generated or analysed during the current study.

## References

[1] Picard RW (2016). Automating the recognition of stress and emotion: from lab to real-world impact[J]. IEEE Multi-Media.

[2] Warsi LQ, Khurshid K. (2022). The Role of Self-Assessment in English Language Teachers’ Professional Development in Pakistan. Education Research International, 2022.

[3] He Z, Li Z, Yang F (2020). Advances in multimodal emotion recognition based on brain-computer interfaces[J]. Brain Sci.

[4] Wang Chuanyu L, Weixiang C (2021). Multimodal emotion recognition based on acoustic and video images[J]. Comput Eng Appl.

[5] Huang HP, Hu ZC, Wang WM (2020). Multimodal emotion recognition based on ensemble convolutional neural network[J]. IEEE Access.

[6] Ding, Mingdu (2020). Li Lin. Facial expression recognition based on CNN and hog dual channel feature fusion[J]. Inf Control.

[7] Zhou S, Huang D, Liu C, Jiang D (2022). Objectivity meets subjectivity: a subjective and objective feature fused neural network for emotion recognition. Appl Soft Comput.

[8] Huang Chengwei J, Zan W, Qingyun (2010). Multimodal emotion recognition based on acoustic signal and ECG signal[J]. J Southeast Univ.

[9] Geetha AV, Mala T, Priyanka D, Uma E (2024). Multimodal Emotion Recognition with deep learning: advancements, challenges, and future directions. Inform Fusion.

[10] Chen Pengzhan Z, Xin X, Fangping (2017). Bimodal emotion recognition based on acoustic signal and text information[J]. J East China Jiaotong Univ.

[11] Wu Z, Pan S, Chen F. A comprehensive survey on graph neural networks[J]. IEEE Trans Neural Networks Learn Syst, 2020: 1–22. 10.1109/TNNLS.2020.2978386 32217482

[12] Cao Zhengfeng. Optimization of random forest algorithm[D]. Capital University of economics and business; 2014.

[13] Lin S, Jinyan X, Mingyue Y (2018). A review of emotion Recognition using physiological Signals[J]. Sensors.

[14] Chew SL, Cerbin WJ (2021). The cognitive challenges of effective teaching[J]. J Econ Educ.

[15] Tremblay-Wragg É, Raby C, Ménard L, Plante I (2021). The use of diversified teaching strategies by four university teachers: what contribution to their students’ learning motivation? [J]. Teach High Educ.

[16] Kee CE (2021). The impact of COVID-19: graduate students’ emotional and psychological experiences [J]. J Hum Behav Social Environ.

[17] Challob AI (2021). The effect of flipped learning on EFL students’ writing performance, autonomy, and motivation [J]. Educ Inform Technol.

[18] Bai S, Hew KF, Huang B (2020). Does gamification improve student learning outcome? Evidence from a meta-analysis and synthesis of qualitative data in educational contexts [J]. Educational Res Rev.

[19] Munir H, Khan EA, Afzal A, Kiran MA. Relationship between Teacher Student Interaction and Student Academic achievement at College Level[J]. Ilkogretim Online, 2021, 20(2).

[20] Lu S, Liu M, Yin L, Yin Z, Liu X, Zheng W. (2023). The multi-modal fusion in visual question answering: a review of attention mechanisms. PeerJ Comput Sci, 9, e1400. 10.7717/peerj-cs.1400 PMC10280591 37346665

[21] Zhang S, Tong H, Xu J (2019). Graph convolutional networks: a comprehensive review[J]. Comput Social Networks.

[22] Lu Guanming H, Jiali Y, Jingjie (2016). A convolutional neural network for facial expression recognition[J]. J Nanjing Univ Posts Telecommunications.

[23] Fisher RA (2012). The use of multiple measurements in taxonomic problems[J]. Ann Hum Genet.

[24] Huang F, Zhang X, Zhao Z, Xu J, Li Z (2019). Image–text sentiment analysis via deep multimodal attentive fusion [J]. Knowl Based Syst.

[25] Truong QT, Lauw HW, Vistanet. Visual aspect attention network for multimodal sentiment analysis[C]. In Proceedings of the AAAI conference on artificial intelligence, 2019, 33(01) 305–312.

[26] Han W, Chen H, Gelbukh A, Zadeh A, Morency LP, Poria S. Bi-bimodal modality fusion for correlation-controlled multimodal sentiment analysis[C]. In Proceedings of the 2021 International Conference on Multimodal Interaction, 2021, 6–15.

[27] Zhou J, Zhao J, Huang JX, Hu QV, He L. Neurocomputing. 2021;455:47–58. MASAD: A large-scale dataset for multimodal aspect-based sentiment analysis [J].

[28] Zhang Z, Wang Z, Li X, Liu N, Guo B, Yu Z. ModalNet: an aspect-level sentiment classification model by exploring multimodal data with fusion discriminant attentional network [J]. Volume 24. World Wide Web; 2021. pp. 1957–74.

[29] Gu D, Wang J, Cai S, Yang C, Song Z, Zhao H, Wang H (2021). Targeted aspect-based multimodal sentiment analysis: an attention capsule extraction and multi-head fusion network [J]. IEEE Access.

[30] Yang H, Zhao Y, Qin B. Face-Sensitive Image-to-Emotional-Text Cross-modal Translation for Multimodal Aspect-based Sentiment Analysis[C]. In Proceedings of the 2022 Conference on Empirical Methods in Natural Language Processing, 2022, 3324–3335.

[31] Song Xujing. Research on multimodal emotion recognition based on text, acoustics and video[D]. Shandong University; 2019.

[32] Kalra P, Sharma V. Mental stress assessment using PPG signal a deep neural network approach[J]. IETE J Res, 2020: 1–715.

[33] Zhang Ting. Research on emotional Acoustics based on pad three-dimensional emotion model[D]. Taiyuan University of technology; 2018.

